# From Anonymous to Public Enemy: How Does a Mosquito Become a Feared Arbovirus Vector?

**DOI:** 10.3390/pathogens9040265

**Published:** 2020-04-05

**Authors:** Didier Fontenille, Jeffrey R. Powell

**Affiliations:** 1 MIVEGEC unit, Université de Montpellier, Institut de Recherche pour le Développement (IRD), CNRS, BP 64501, 34394 Montpellier, France; 2 Department of Ecology and Evolutionary Biology, Yale University, 21 Sachem Street, New Haven, CT 06511-8934, USA; jeffrey.powell@yale.edu

**Keywords:** mosquito, culicidae, *Aedes aegypti*, *Aedes albopictus*, emergence, arbovirus

## Abstract

The past few decades have seen the emergence of several worldwide arbovirus epidemics (chikungunya, Zika), the expansion or recrudescence of historical arboviruses (dengue, yellow fever), and the modification of the distribution area of major vector mosquitoes such as *Aedes aegypti* and *Ae. albopictus*, raising questions about the risk of appearance of new vectors and new epidemics. In this opinion piece, we review the factors that led to the emergence of yellow fever in the Americas, define the conditions for a mosquito to become a vector, analyse the recent example of the new status of *Aedes albopictus* from neglected mosquito to major vector, and propose some scenarios for the future.

## 1. Introduction

In a time of major social, climatic and environmental changes, several old concepts are back in fashion: “health is one” (one health approach), “the microbe is nothing, the context is everything” (Antoine Béchamp, Louis Pasteur), “diseases will always continue to emerge” [1]. All these old, but still very relevant views require a holistic approach, taking into account the complexity of interactions between diseases, microbes, hosts, vectors, environment, and their evolution as described by Mirko Grmek [2], under the term pathocenosis. Emergence of SARS-Cov2 viruses (Covid-19 disease) in 2019, dramatically confirms these predictions.

It is generally accepted that, other than our own species, the “most dangerous animal in the world” for humankind is the mosquito. This figure of speech, however forceful it may be, is misleading. Yes, there are mosquitoes responsible for the transmission of major human pathologies (malaria, filariasis, yellow fever, dengue, Zika, chikungunya, haemorrhagic fevers, encephalitis, etc.), and it is likely that most blood-sucking mosquitoes transmit some pathogens to some vertebrates. However, given that only a small minority of the thousands of mosquito species account for the vast majority of human diseases, “the mosquito” should be afforded the principal of “*habeas corpus*”, innocent until proven guilty.

In this opinion piece, we discuss the factors that lead to the emergence of a vector in the human environment causing the transmission of viruses pathogenic to humans. A related, but distinct phenomenon which we do not discuss, is the establishment of newly introduced pathogenic viruses in sylvatic vectors, leading to new cycles of enzootic transmission.

## 2. Vector or not yet Vector: Guilty or Presumed Innocent

Two species of mosquitoes of particular relevance to human public health, *Aedes aegypti* and *Aedes albopictus*, are good models for understanding, estimating and, if possible, anticipating and limiting the risk of emergence, establishment and transmission of pathogens to humans by vectors [3,4]. *Aedes aegypti* was suspected to be a vector of the yellow fever virus by Beauperthuy and later Finlay since the middle of the 19th century [5,6], and experimentally demonstrated as a vector in 1900 by Reed et al. [7], and therefore an enemy to be killed [8,9]. In contrast, *Ae. albopictus* became widely known only over the last 40 years when it was gradually detected on all continents (except Antarctica) outside its native region of Southeast Asia and implicated in the recent chikungunya pandemic [10,11]. These two species (*aegypti* and *albopictus*) of genus *Aedes* and subgenus *Stegomyia* are responsible for virtually all major outbreaks of diseases caused by the major primate arboviruses: dengue, Zika, chikungunya, and yellow fever. Both mosquitoes are invasive species, spreading thanks to man-made means of transport, and are extremely well adapted to human environments.

Transmission of viruses to humans by mosquitoes seems to be the rule for many public health officers. Actually, it is an exception. The vast majority of mosquitoes do not transmit human diseases. Of the 3585 species of mosquitoes currently formally described, less than 30 transmit the yellow fever virus, either naturally or in the laboratory. In other words, more than 3500 species of mosquitoes do not transmit the yellow fever virus. They are not guilty. There are many reasons for this: (1) the virus is not present in the geographical area of the vector mosquito species; either by “luck” because it was never introduced, or due to the absence of vertebrate amplifying hosts allowing the maintenance of cycles (primates in the case of yellow fever); (2) the mosquito is not competent to transmit (absence of required receptors or innate immunity controlling the pathogen); (3) the biology of the mosquito is not compatible with transmission (life span too short, does not take blood meals from primates, etc.).

However, our present understanding should not be taken as dogma. We must keep an open mind and explore what factors can make a non-vector mosquito become a vector, or what can cause a so-called “secondary” vector to become a major vector for humans. To continue our metaphor, what turns an “innocent” mosquito into a “guilty” menace; then into an effective vector to humans? In theory, it is very simple. Only two conditions are necessary: (1) the mosquito is physiologically capable of sustaining a virus infection that spreads to saliva and takes multiple blood meals from primates, i.e., was always a potential vector, (2) an event or several events occur in its environment that allow it to express this vector potential. Subsequently, adaptive mechanisms, under selection pressure, may cause this mosquito to become responsible for severe outbreaks. However, while this seems simple, in reality, a large number of factors are necessary for the conditions to be met that allow the emergence of a new vector.

## 3. Short History of Yellow Fever: Once Upon a Time in America

The yellow fever virus circulates in African forests from monkey to monkey, transmitted by forest *Aedes* (*Ae. africanus, Ae. luteocephalus, Ae. furcifer, Ae. simpsoni* s.l., *Ae. opok*, etc.), with incursions into villages where domestic anthropophilic *Ae. aegypti* are established, and able to replicate and transmit YF virus [12]. These domestic *Ae. aegypti* are themselves the result of a slow adaptation of forest ancestors to human habitats and blood [13]. The virus, once in humans, following cross species transmission, can be introduced through the movement of humans into African cities, where it finds *Ae. aegypti* populations adapted to urbanisation and able to generate outbreaks.

This village or urban yellow fever virus, already adapted to human and *Ae. aegypti*, was introduced into tropical America, probably via viremic people and/or *Ae. aegypti* transported on ships, during the triangular slave trade, which started in the 16th century [14,15]. Upon arrival to the Central and South American rainforest, not only did the virus find susceptible New World monkeys but also new competent endemic mosquitoes from two genera, *Sabethes* sp. and especially *Haemagogus* sp. Yellow fever virus thus had a second phase of cross species transmission (African monkeys to American monkeys, African Aedes mosquitoes to American mosquitoes).

**Figure 1:**
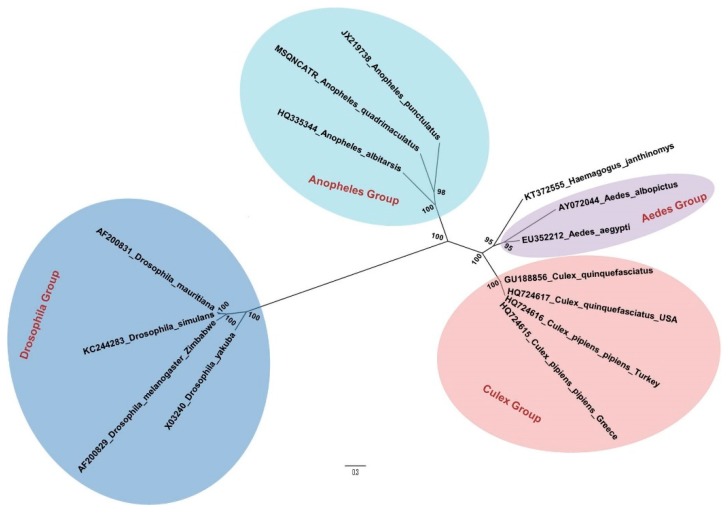
Phylogenetic tree built by the method of maximumlLikelihood, from sequences of mitochondrial genes COX1, COX2, NAD4, NAD5 and CYOB of *Haemagogus janthinomys* and other species of Diptera, from da Silva Lemos et al. 2017 [25]. The bootstrap values are represented in each node.

The evolutionary history of *Haemagogus* genus mosquitoes, which are only found in the New World, is not well known. Phylogenetically, they seem to be quite close to the *Aedes* (Figure 1), which may explain their ability to transmit, more or less, the same viruses as *Aedes aegypti*: yellow fever, dengue [16], Zika [17], and chikungunya [18], all introduced into South America. New cycles of jungle yellow fever have thus developed, involving South American primates and tree-dwelling *Haemagogus* mosquitoes. These cycles have spread throughout South and Central America, where they are still active, as shown by the 2016–2018 yellow fever epidemic in Brazil, with more than 700 deaths [19]. This combination of both a sylvan and an urban cycle for yellow fever in the Americas regularly fuelled epidemics that greatly affected the history of colonization of the New World [20]. As *Ae. aegypti* adapted to American cities, even non-tropical locations were subject to yellow fever epidemics, such as the well-known Philadelphia epidemic in 1793 (5000 deaths). A largely successful eradication program against *Ae. aegypti* began initially in the 1930s and was started by the Rockefeller Foundation [21], and this continued with DDT after World War II. At the same time, wide vaccination coverage was undertaken. This led to almost no outbreaks of yellow fever due to *Ae. aegypti* in the Americas, since about 1970, although *Ae. aegypti* is again abundant [22], and able to transmit dengue fever virus and the recently introduced Zika virus. It is thought that the Brazil 2016-18 yellow fever outbreak was almost entirely due to spill over from the sylvan cycle and involved *Haemagogus* and *Sabethes* mosquitoes [23,24].

Interestingly, endemic yellow fever has never been reported in Asia, Madagascar or other Indian Ocean islands, for reasons that are still poorly understood. Primates (monkeys and humans) from these regions are susceptible, and *Ae. aegypti* and other experimentally competent *Aedes* mosquitoes are present there [26,27,28]. Importantly, *Haemagogus* sp. and *Sabethes* sp. are absent from Africa, the Indian Ocean and Asia.

## 4. The Necessary Conditions

The history of yellow fever leads us to the consideration of the factors necessary, but not always sufficient, for a mosquito species that was initially of little interest to humans, to become a public health problem and an enemy to fight.

First of all, the mosquito, or to be precise, a given population of a given species of mosquito, must be biologically able to transmit the virus. This ability is called vectorial capacity, which includes vector competence (i.e., the ability of a mosquito to become infected after ingestion of an infected blood meal and later transmit the virus via its saliva). Both terms, vectorial capacity and vector competence, have been formalized since MacDonald [29], summarized by Cohuet and coll. [30], and include the following parameters:

- The vector–host ratio (i.e. the vector density in relation to vertebrate host): *m* (the mosquito abundance);
- The human feeding rate: the number of human bites per mosquito, per day: *a* (mosquito - human contact);
- The daily survival rate (i.e. the probability of a mosquito surviving each day): *p* (mosquito longevity);
- The extrinsic development time, the time necessary for viruses to complete development from ingestion in midgut to the saliva: *n*;
- The infectiousness of the mosquito to the vertebrate host: *b* (largely dependent on virus titre in saliva)
- The susceptibility of the vertebrate host to the virus (e.g., immune state, age, health, etc): *c*;
- The vertebrate host infectious period: 1/r (how long the virus titre in the vertebrate remains at a level needed to infect a mosquito);

Knowing the values of these parameters makes it possible to calculate the basic reproductive rate of the virus, *R_0_*.

*R*_0_ = (*ma*^2^ × *p*^*n*^ / − ln *p*) × *bc* × 1/*r* (1)

*R_0_* is the total number of cases derived from one infective case that the mosquito population would distribute to vertebrate hosts. *R_0_* must equal at least 1 for the disease to persist or spread. For values less than 1, the disease will go extinct.

However, while we can write the simple equation above, in reality the parameters (*m, a, p, n, b, c, r*) are themselves complex, being dependent on many other factors. For just the mosquito, a non-exhaustive list includes mosquito genotype, mosquito microbiome including viruses, integrated RNA virus sequences in mosquito genomes, predation, competition at larval and adult stage, previous exposure to related viruses, etc. [31,32]. Moreover, it is not yet known which receptors in the cells of the stomach and salivary glands of mosquitoes are involved in the entry and exit of flaviviruses, like yellow fever virus [33]. 

**Figure 2:**
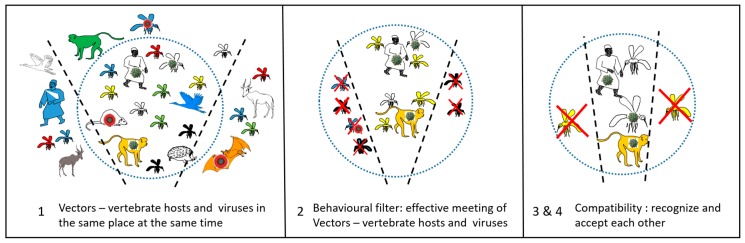
Encounter and compatibility filters, from Euzet and Combes [34].

Just this intrinsic biology of the mosquito is not sufficient to make it a vector. Its environment may, or may not, allow this vector potential to manifest itself. According to Euzet and Combes [33], the specificity of the vector–pathogen interaction passes through four stages, which they named encounter and compatibility filters (Figure 2). These four stages are: (1) to co-occur in space and time, (2) to meet each other (behavior), (3) to recognize each other (receptors), and (4) to accept each other (immunity).

All these conditions are rarely met, and it is therefore understandable why being a vector is an exception. However, considering the very large number of mosquito species and large number of viruses, while we are presently facing only a limited number of dangerous cycles, this must be a very small fraction of thousands, if not millions, of other potential cycles that have failed. The understanding of the current efficient cycles allows us to conceive possible future cycles.

We can hypothesize that only a few species in the genera *Aedes*, *Haemagogus*, and *Sabethes* have the capacity to replicate and transmit yellow fever virus to primates, i.e. (1) possess the receptors for cellular penetration of the virus, (2) have insufficient innate immunity to suppress virus replication, (3) live in the same environments as viremic vertebrates (monkeys or humans), (4) take blood from these vertebrates, and (5) survive long enough to retransmit the virus.

## 5. *Aedes albopictus*: From Local to Global Concern

*Aedes albopictus* became widely known only over the last 50 years, when it was gradually discovered on all continents outside its native Asia and caused a chikungunya pandemic [10,11,35,36]. A total of more than 40 viruses can be transmitted naturally or experimentally by *Ae. albopictus* [36]. Experimentally, it will take blood from many vertebrate species [37]. It also lives longer than *Aedes aegypti* [38]. *Aedes albopictus* therefore perfectly fulfils the necessary conditions in terms of biology and events to be an excellent vector of virus to humans.

*Aedes albopictus* is considered to be phylogeographically native to Southeast Asia [10]. This assumption is based on the fact that this mosquito is present everywhere in the forested areas of this region and that many species close to the Albopictus subgroup, member of the Scutellaris group, are present in Southeast Asia. However, the notoriety of *Ae. albopictus*, compared to relatives, comes from the fact that it has moved out of its area of origin, becoming worldwide in 50 years, adapting perfectly to urbanization, temperate climates, and international transport, as well as being involved in several dengue and chikungunya epidemics. 

In tropical Southeast Asia, the five morphologically closely related species of the Albopictus subgroup live in forests (*Aedes novalbopictus*, *Aedes patriciae*, *Aedes seatoi*, *Aedes subalbopictus* and *Aedes pseudalbopictus*) and lay their eggs in tree holes and bamboo stumps [39,40]. These species are likely to be vectors of arboviruses to vertebrates from which they take blood. These forest-confined viruses are largely unknown, but may emerge through increased contact with humans or domestic animals (see below). They could then be transmitted by domestic vectors, such as *Ae. aegypti* and *Ae. albopictus*, in a pattern similar to the emergence of the yellow fever virus in Africa from forests to villages [12].

In Laos, *Ae. albopictus* is reported from deep natural forest [41], rubber forest and secondary forest [42], as well as in towns and villages. In contrast, in neighbouring countries, such as in Cambodia, this mosquito was found along hundreds of meters of forest edge, whereas *Ae. pseudalbopictus*, is found in deeper natural forests. *Aedes albopictu*s is frequently found in cities such as the capital, Phnom Penh (*Boyer pers*. *com.*). Similarly, in Malaysia, *Ae. albopictus* is rare in forests [43], and in Yunnan Province, China, *Ae. albopictus* is sometimes more abundant than *Ae. pseudalbopictus* in bamboo forests, but is often absent from deep forests [40].

Outside Asia, *Ae. albopictus* populations display highly variable success in invading the deep forest. In Brazil, where *Ae. albopictus* was observed for the first time in the 1980s, Pereira dos Santos et al. found that *Ae. albopictus* was able to enter degraded forest in the Manaus region, up to 750 metres from the edge [44]. In Gabon, where *Ae. albopictus* was first reported in 2007, it was captured 12 years later in the Lopé natural forest, several kilometres from villages or clearings (*Paupy pers. com*.).

In both its native range Asia and recently invaded South America and Africa, *Ae. albopictus* has retained its ancestral capacity to colonize forest environments, laying eggs in natural pools of water and taking blood from nondomestic vertebrates. It is logical to think that under these conditions, *Ae. albopictus* populations would take blood from vertebrates carrying as yet unknown viruses, such as from monkeys, terrestrial mammals, birds or reptiles. If these forest-breeding *Ae. albopictus* are able to replicate these viruses, and then retransmit them to humans, they would then be excellent bridge vectors, allowing the emergence of forest viruses hitherto confined to sylvatic *Aedes*-vertebrates cycles. We can speculate that this is most likely to occur in Asia. In Africa, *Ae. aegypti* has already brought many, perhaps most, human adapted viruses from the forest to human habitats, such as yellow fever, dengue, chikungunya, and Zika viruses [13].

Mogi et al. [40], suggest that the spreading of *Ae. albopictus* from its original tropical forest region was possible following evolution from an ancestral wild species, due to adaptation to man-made habitats and then migration with humans to temperate climate regions, where *Ae. albopictus* developed a winter diapause. In most introduced localities, it probably encountered only limited competition from native mosquitoes and when it did, *Ae. albopictus* proved to be a robust competitor, e.g., with *Ae. aegypti* [45]. It is likely that the ancestral wild species was already a vector of forest vertebrate arboviruses, for example, dengue-like flaviviruses, or chikungunya-like alphaviruses, which are monkey viruses [46].

From the foregoing, it appears that *Ae. albopictus*, while being ancestrally a forest-breeding mosquito like its close relatives, among these relatives it is the most closely adapted to forest margins (ecotone), the transition from forest to degraded or secondary forests, open grasslands or scrub. *Aedes aegypti* in Africa is similar [47]. This may have pre-adapted these two *Aedes,* among all their congeners in Asia and Africa, to come into contact with human settlements and thus to adapt to this new niche, human settlements. 

From regions recently colonized by *Ae. albopictus*, “modern” populations, highly adapted to the urban environment and able to transmit viruses such as chikungunya, Zika and dengue, spread to all continents. It is likely that invasive populations re-invaded regions where ancestral populations already existed, such as in Asia and the Indian Ocean. These ancestral populations then found themselves in unfavourable competition and modern *Ae. albopictus* replaced them [48].

It is not too difficult to predict the epidemiological future of *Ae. albopictus*. Its geographical distribution will increase, especially in temperate regions, its control by insecticides will be more difficult due to the emergence of resistance, and human pathogenic viruses will adapt to this new vector, increasing its efficiency to transmit. *Ae. albopictus*-vectored epidemics are almost certain to increase. It could be responsible for the epidemics of yellow fever, or of currently unknown viruses transmitted by forest edge *Aedes* mosquitoes of South American, South East Asia or Central Africa. A major unknown for *Ae. albopictus’* impact in temperate regions is whether arbovirus replication at lower temperatures is selected to be rapid enough to reach saliva before the female dies.

## 6. Scenarios for the Future: The Worst Doesn’t Always Happen

To quote Charles Nicolle [1], it is certain that new epidemics will appear. Mosquitoes and viruses are poised to ambush humans following changes in vector ecosystems [49]. The process of establishment may be abrupt and rapid, as was the recent Zika pandemic with *Ae. aegypti* as vector, or more gradual, going through adapting mechanisms, as was the case with the emergence of the West Nile virus in several temperate cities, particularly in the USA [50].

The optimistic view, but not very realistic, is that the worst is already behind us. Urban environments, where the majority of the humans now live, are already colonized by *Ae. aegypti*, *Ae albopictus*, and/or *Culex pipiens*, which transmit or can transmit known viruses of tropical or equatorial origin: dengue, chikungunya, Zika, yellow fever, West Nile, as well as, at least potentially, many other tropical viruses like Japanese encephalitis and Rift Valley fever, with variable success depending on species and environments [13]. However, this optimistic view needs to be tempered. With global changes (environment, climate, demography, movements), the distribution areas of some of these human adapted vectors and viruses will continue to expand and we will see a steady increase of epidemics of viruses, including in temperate regions. While we cannot predict details of time and place, we know it will happen, and we can prepare for it.

A more pessimistic view is that the reservoir of unknown forest viruses, not presently affecting humans, but potentially transmissible to domestic animals and humans, is immense in Asia, Central and South America, and Africa. More than 500 arboviruses have so far been identified and described, but this is only the tip of the iceberg. From about 1930–1980, the Rockefeller Foundation sponsored a repository for viruses from field-collected arthropods, primarily mosquitoes, that reached more than 4000 isolates of insect-specific viruses or arboviruses, the vast majority of which are undescribed [51], and even this collection is far from complete. Consequently, several non-exclusive scenarios for the emergence of new human viral epidemics are possible:

(1) Via primate-biting bridge vectors such as *Ae. albopictus* from forest edges in South America, Africa and Asia, forest *Aedes* of the Albopictus group in South East Asia, *Haemagogus* in South America, *Stegomyia* from forest galleries in Africa. In South America, *Haemagogus* and *Sabethes* are likely to transmit any new potential human viruses among primates and, as displayed by yellow fever, if these viruses are capable of transitioning to transmission in human habitats, it is likely to have already occurred.
(2) By misfortune, as happened with the establishment of a sylvan cycle of yellow fever in Central and South America 500 years ago, new viruses may appear in transmission cycles that are heretofore unknown. The increase in trade and travel, and the establishment of invasive species (such as *Ae. albopictus*, *Ae. koreicus*, and *Ae. japonicus*, and even *Ae*. *aegypti* in Europe) suggest that this risk should not be overlooked.
(3) Via increased human contacts with wild cycles due to deforestation and irrational forest exploitation. These ecological modifications favour the emergence of viruses from forest edges, and then to the human environment.
(4) Via zoonotic cycles. For example, many viruses (West Nile, Japanese encephalitis, St Louis encephalitis virus, Murray valley, usutu) which are bird viruses, can be transmitted from birds (or mammals such as pigs) to humans, via vectors taking blood meals from both birds and humans, such as *Cx. tritaeniorhynchus* or *Cx. pipiens*, *Cx. quinquefasciatus.* It is very likely that some of these viruses, presently confined to wild cycles, will emerge in the coming years somewhere in the world, as a result of socio-ecological changes. Their shift from endemic to epidemic will be facilitated by close contact between humans and vertebrate hosts (urban commensal birds or rodents, farm animals).
(5) By opening an ecological niche in urbanized areas. It is conceivable that local effective vector control by way of elimination of *Ae. aegypti* or *Ae. albopictus* would open their ecological niche in some areas, allowing a new species, such as *Ae. malayensis* in South-East Asia, already recognized as a vector of dengue and chikungunya viruses, to begin to colonize even closer to human populations. This scenario has not happened yet. *Aedes albopictus* may have replaced *Ae. aegypti* or vice versa, but for the moment no third species, with high vectorial capacity, has occupied their niches.
(6) Via the evolution of viruses already known, but which have not yet found the conditions for emerging and spreading. Genetic changes in virus strains could lead to better adaptation to new vectors and a better transmissibility, as happened with the chikungunya virus and *Ae. albopictus* [52]. Viruses may also evolve resistance to drugs, when any are used, or human immune defences. Given the short generation time, large population size, and high mutation rate of RNA viruses (like yellow fever, dengue, and most pathogenic arboviruses), virus adaptation to efficient transmission by a human-preferring mosquito is rapid. That is, the virus more readily adapts to the mosquito (and vertebrate host), not the mosquito to the virus [53].

Among the hundreds of wild animal viruses, several viruses are regularly cited as worthy of surveillance. This is the case of the Mayaro alphavirus, closely related to the chickungunya virus. It is a South American virus of mammals, including monkeys, mainly transmitted by *Haemagogus* mosquitoes, and which could adapt to urban *Ae*. *aegypti* or *Ae. albopictus*, or to some other anthrophilic Culicidae. However, these other mosquitoes are considered poor vectors [54,55]. In Africa, the Spondweni virus is genetically close to the Zika virus, which places it on the list of viruses to keep an eye on. While it is indeed pathogenic to humans, very few human cases have been detected; it has no known domestic cycle. It is poorly transmitted experimentally by *Aedes (Stegomyia)*, being efficiently transmitted by zoophilic mosquitoes (*Aedes circumluteolus*, *Mansonia africana*), but this situation could change [56]. Indeed, this virus has already been found in Haiti [57] in *Cx. quinquefasciatus,* a human-biting mosquito recorded in all tropical regions.

## 7. Conclusions

On a final note of optimism, it is expected that new outbreaks can be detected more quickly, thanks to surveillance and warning systems that have improved greatly in recent decades and thanks to the development of efficient, rapid and less costly diagnostic techniques. Moreover, not all emergences will find favourable environments, including encounter and compatibility filters, and are likely to quickly die out. For public health authorities to succeed in limiting new outbreaks, The International Health Regulations, published in 2005 by WHO [58], provide a framework and obligations for member states, and give hope that new epidemics could be brought under control through early detection, efficient vector control, vaccination when available, and other public health measures. While worst case scenarios should be planned for, they seldom actually arise. However, they happen regularly and the recent Covid-19 pandemic, which was not caused by an arbovirus, reminds us that, although we are aware that this can happen, we are not always properly prepared.
